# Tics in Children and Their Treatment

**Published:** 1910-07

**Authors:** Henry C. Whelchel

**Affiliations:** Douglas, Ga.


### TICS IN CHILDREN AND THEIR TREATMENT.

DR. HENRY C. WHELCHEL, DOUGLAS, GA.

In presenting this subject to-day, it is not for the purpose of giving you something new, but to call attention to a condition that we general practitioners too often treat lightly, by telling the mother that it is a habit, and the child will out-grow it.

It has been said, that the frequency of tics is in direct proportion to the civilization of a people. Considering the frequency of this condition, and the discomfort which it causes the patient and his family, it must be said that the subject has not received the attention which it deserves.

Many of the tics, which are so annoying and obstinate in adult life, give as their outward manifestation a tic in early life. Often such a tic was neglected, looked upon as a bad habit, to be out grown, or treated with drugs without relief, and the patient and his family discouraged. It cannot be too strongly emphasized that a tic is simply a motor manifestation of a peculiar mental state, and should be looked upon as a warning, a danger signal of possible trouble to come.

Tic, some times called mimic spasm, habit spasm, etc., has been defined by Gowers as "A spasmodic movement, half voluntary in aspect, which the patient is unable to control."

By Guinon as "An habitual, and conscious movement, resulting in the contraction of one or more of the muscles of the body; reproducing most frequently in an abrupt manner; some reflex or automatic action of common life." By Meigs and Feindel: "A tic is a psychomotor affiection in which two elements are essential: First, an abnormal state, a lack of control of the will; second, a motor manifestation due to some stimulus from without, or from the brain."

When the stimulus acts from without, the movement is executed with a definite purpose.

By repetition this movement becomes habitual or automatic; continuing without cause or purpose, the will being too weak to inhibit it.

The original purpose of the movement, or posture, is usually apparent; it is a co-ordinate act, adapted to some definite object; as for example, a gesture of defense, or an expression of some emotion, as of grief, pain, fright or joy. These movements are repeated in response to an irresistable desire, and are followed by a feeling of satisfaction. These tics of which blepharospasm is a type, are more or less under the control of the patient, who can by an effort of the will do considerable to repress them, but such repression is followed by a vague feeling of discomfort.

The contractions follow each other at irregular intervals, and are rapidly executed, and may be repeated at great rapidity from two, to many times, when a lull occurs for an indefinite time. Any excitement or embarrassment promptly recalls, and intensifies the morbid motions.

On the other hand, and decided interest fixing the patient's attention, interrupts the twitchings. They also disappear in sleep. There are no sensory disturbances.

This affection is by no means rare. In the physical examination of a large number of children in the New York public schools, it was found that about one in eighty was affected with muscular tic.

In the etiology of this affection, as in all the neuroses of predisposition, the favorable soil; second, the environment, including the method of training. Both sexes are about equally affected. In this it differs from Chorea, in which more than twice as many cases occur in females. Tics rarely manifest themselves before the fourth year, most commonly observed about the seventh or eighth year.

In predisposed individuals, slight things may act as exciting causes, such as physical or mental shock, traumatism or fright, infectious diseases, by lowering the vitality.

Frequently the movement or gestures was originally performed for a definite purpose, for example: a child has a granular conjunctivitis, or a foreign body in the eye, blinking is done with an endeavor to remove the cause of irritation, as the neck is scratched by the collar, and the finger is run around the neck to separate it. In the normal child as soon as the source of irritation is removed the act is unnecessary, but not so with the future victim of a tic. Here the act continues in response to that irresistable impulse. Those cases becoming more widespread involve the neck and upper extremities, so that attitudes and gestures are produced in conformity with the underlying mental idea. I will mention only a few of the more common forms of tics. One is the blinking of the eyelids or blepharospasms, which may occur with other movements of the face, hands or shoulders. Another is the licking of the lips, and another which is often met with, is the long drawn inspiration, with or without a sigh. Such children will some times complain of a heaviness on the chest, and can't catch their breath. On examination no true dyspnea is found, and the chest shows no pathological changes. The quick nod so frequently seen in girls, perhaps originated from a desire to adjust their hats.

Another form which is very important, on account of the proper treatment, is the so-called "Mental Torticollis," in which some deviation of the head is customary, and is spasmodically maintained. Ordinarily it ceases when the patient lies down, or it can be controlled by a slight amount of manual pressure upon the head or face.

Spasmodic sniffing of the nose is another form, originally caused from adenoids, or acute nasal catarrh. Still another quite common form, is face grimaces, commonly called sun grins.

### TREATMENT.

Though not dangerous to life, few affections are more troublesome to their victims than the twistings, and twitchings of muscles, comprised in the above title. As the patients grow older, they are willing to take great pains to get rid of the unpleasant gestures, which though not painful in themselves, cause a great deal of moral suffering, besides interfering with efficiency, both in business and social life.

The obstinacy of these afflictions has caused them to be looked upon with despair by practitioners; but the medical pessimism with regard to them is not well founded, for they are in reality ameanable to treatment. If there are abnormalities near the site of the muscles affected, these should receive attention.

Conjunctivitis, especially the granular form, should be treated, errors of refraction corrected, adenoids removed, etc.

However, these can never be more than contributing causes, for the neurosis, the mental condition is always the essential factor.

Given the proper psychical peculiarities, almost any form of irritation may act as an exciting cause. Still, I believe it was, who pointed out, that a tic may become more pronounced, at least for a time after an operation, probably from the mental shock; therefore for "Mental torticollis" he says, "instead of operating on the patient, and advising muscular exercises for months after the wound has healed, it is better to try the effect of the exercise for months or even years, before deciding on the operation at all."

Drugs, except tonics, are in most cases useless, unnecessary, and contraindicated. Electricity, hypodermatic injections act primarily in a mental way; others such as hydrotherapy, massage, and tonics by improving the general health.

Of great importance is the regulation of the diet, and the mode of life. The patient should take mild exercise in the open air, but not to the point of fatigue. He should go to bed early, and should not engage in any excitement in the evening. In the mild cases, the discipline, regularity, punctuality, and attention required in schools are advantageous. But not so with severe cases, they should have a complete change of surroundings, with cessation of mental efforts. All the foregoing measures though valuable, are not sufficient themselves to effect a cure, and are only aids to the real treatment which is educational.

The method as originated by Herrman, is simple, and its very simplicity renders it possible for the patient and parents to carry out the treatment to a great extent themselves.

It consists of two parts: First, immobilization primarily of the affected parts. Second, active exercise of the affected parts.

The patient is seated before a mirror, the physician standing behind the patient, directs him to remain perfectly quiet, like a statute for a stated time. This has for its object immobilization of the parts affected, and gives to the patient an occular demonstration that he can control the movements, if he only wills so. This he is required to do for a longer, and longer time.

The second part of the treatment consists of active exercise of the muscles affected. For the eyes, opening and closing, first together, and then separately. For the mouth and lips, opening and closing alone, and then combining with the opening and closing of the eyes.

Reading aloud, recitations and singing. For the movements of the head, flexion and extention, looking first up, and then down, followed by lateral movements. All movements should be performed slowly, and regularly at command. Other authorities have advocated respiratory exercises. Letting the patient stand against a wall, his arms at his sides, his shoulders thrown backwards like a soldier, taking first a long, deep inspiration, at the same time raising the shoulders, and then a long expiration, dropping the shoulders. Such exercise is especially valuable in respiratory tic, and recommended by Hammond for stammering.

I had the opportunity during my twelve or thirteen years of service as surgeon of the N. G. A. College, of watching the effect of a military training on three cases of muscular tic. Two were completely cured, the third left college after one year, hence I don't know the result in his case.

We should remember there is a tendency for these spasms to return, therefore the patient should be kept under observation for a long time, and advised to persist in treatment, remembering that the reward of victory is cure.

References—Gowers, Guinon, Meigs & Feindel, Herrman, Hammond, Church-Peterson, Williams, Caille, Kerley.

